# An Examination of Responses to COVID-19 Contact-Tracing Efforts in Black/African American and Hispanic/Latinx Communities of Los Angeles

**DOI:** 10.1089/heq.2023.0243

**Published:** 2024-08-07

**Authors:** Sharon Cobb, Katrina Schrode, Hafifa Siddiq, Shanika Boyce, Kelly D. Taylor, Roberto Vargas, Nina Harawa

**Affiliations:** ^1^ College of Nursing, Charles R. Drew University of Medicine and Science, Los Angeles, California, USA.; ^2^ College of Medicine, Charles R. Drew University of Medicine and Science, Los Angeles, California, USA.; ^3^ David Geffen School of Medicine, University of California, Los Angeles, California, USA.; ^4^ Institute for Global Health Sciences, University of California, San Francisco, California, USA.; ^5^ Health Services Research and Policy Core, Urban Health Institute, Charles R. Drew University of Medicine and Science, Los Angeles, California, USA.; ^6^ David Geffen School of Medicine and Fielding School of Public Health, University of California, Los Angeles, California, USA.

**Keywords:** COVID-19, contact tracing, Hispanic or Latino, public health, Black or African American, communicable diseases

## Abstract

**Objectives:**

To investigate the experiences and perceptions of COVID-19 contact-tracing efforts among cases tested in under-resourced and predominately Latino and Black communities of South Los Angeles, California.

**Methods:**

Study involved a cross-sectional survey with 1,713 adults. Recruitment occurred between June and November 2021 with eligible individuals who had previously received a COVID-19 diagnosis through designated testing sites. The LA County Department of Public Health operated a culturally responsive program for contact tracing that included provision of education and service referrals to newly diagnosed cases through much of the pandemic.

**Results:**

Participants were majority female (63%), Hispanic/Latino/a/x (64%), ages 18–40 (69%), and surveyed in English (77%). Overall contact-tracing experiences were rated positively, regardless of demographics (average means of 3.1–3.2/4.0). Those surveyed in Spanish were more likely to endorse positive statements if their contact tracer also spoke Spanish. Although over 75% of participants shared a range of the different information types requested, 49–52% endorsed concerns about data security and uses of the solicited information.

**Conclusions:**

Despite eliciting some concerns, contact-tracing efforts were generally positively received.

**Policy implications:**

Investments in contact tracing in similar communities should consider language-concordant contact tracers, community-based health worker training in trust building, and addressing social and health needs.

## Introduction

Coronavirus disease 2019 (COVID-19) emerged as one of the most challenging global health crises since the 1918–1919 Influenza Pandemic.^1–3^ To respond, public health systems in the United States rapidly implemented case investigation and contact tracing to isolate COVID-19 cases and identify potentially exposed individuals for quarantine.^4–7^ Although some research has been undertaken to examine these efforts,^5,8^ the pandemic’s overwhelming toll, coupled with polarization over many public health control efforts, underscores the need to examine experiences with contact-tracing programs among disproportionately affected racial/ethnic groups, including African American/Black and Hispanic/Latino populations.^9,10^

Contact tracing, a containment strategy to identify potential sources of infection, employs identification, tracking, and testing of individuals who were likely exposed to the index case during their infectious period.^11,12^ Implementing contact tracing is resource intensive and time-consuming, leading many to question its value given the already strained public health system.^13–15^ Nevertheless, studies indicate that the lack of robust testing and contact-tracing programs contributed to COVID-19 resurgences.^16,17^

Surveillance estimates indicate two-thirds of individuals diagnosed with COVID-19 in 2020 were either not contacted or did not provide any potential contacts for COVID-19 tracing.^4^ A 2020 examination of contact-tracing completion rates found 48% of 5,514 COVID-19-positive cases attempted in a North Carolina County did not provide any contacts.^5^ Contact-tracing programs have been critically understaffed because of workforce shortages. Approximately 70,000 tracers were employed in the United States in 2020, well below the national goal of 100,000 tracers.^18^ Technological solutions to reduce the personnel burden are being rapidly implemented, and the acceptability of mobile contact-tracing applications is rising.^19–24^

### Minorities and Contact Tracing

Age-adjusted COVID-19 infection, hospitalization, and mortality rates were elevated in African American/Black, American Indian, and Hispanic/Latino groups compared with non-Hispanic/Latino Whites and Asian ethnic groups across the United States.^25–27^ During the time period of this current study, data within Los Angeles County showed that African American/Black and Hispanic/Latino individuals had mean age-adjusted COVID-19 case rates of 212/100,000 and 392/100,000 people, respectively, whereas White individuals had age-adjusted case rates of 158/100,000. Moreover, White individuals had an average death rate of 3/100,000, which was observed as doubled for African American/Black individuals, and over three times for Latino individuals. Evidence points to low uptake of contact tracing in African American/Black and Hispanic/Latino communities. Shelby et al. (2021) revealed African Americans/Blacks were less likely than non-Hispanic/Latino Whites to be interviewed for COVID-19 contact tracing in Connecticut.^7^ Miller et al. (2021) found that just 32% of 3,752 cases interviewed in five predominately Hispanic/Latino counties in Washington State reported close contacts in the state’s contact-tracing program.^8^ Various studies describe structural and attitudinal barriers to participation in public health initiatives.^28,29^ Barriers for COVID-19 prevention, testing, and contact tracing include medical mistrust, lack of culturally sensitive messaging, misconceptions, privacy concerns, and conflicting public health messages.^30–34^

The U.S. response to the COVID-19 pandemic has been complicated by the social, economic, criminal justice, and medical inequities experienced by racial/ethnic minority communities.^35–37^ Therefore, increased contact-tracing efforts may have been perceived as excessive surveillance.^38^ To date, there is no published research on how racial/ethnic minorities perceived contact-tracing efforts outside of immigration-related ramifications.^39,40^ This study investigated experiences of and confidence in contact-tracing efforts among populations who tested positive for COVID-19 in under-resourced and predominately African American/Black and Hispanic/Latino areas of Los Angeles, California. All individuals who tested positive should have been contacted by the Los Angeles County Department of Public Health (LAC-DPH) or contacting agencies whose tracers were trained by LAC-DPH to conduct contact tracing during the study period.^41^ In addition to LAC-DPH public health specialists, disaster service workers, state, and LA City staff, including librarians and public works officials, were trained as contact workers. Emphasis was placed on expanding the cultural and linguistic competency of the contact-tracing workforce. From the inception of the COVID-19 pandemic to April 2022, contact tracers completed over 711,000 interviews with 30.7% of identified cases and over 187,000 interviews with 63% of identified close contacts.

Our research team based at Charles R. Drew University used a cross-sectional design to survey participants about being contacted by the LAC-DPH for contact tracing and their perceptions of this public health effort. To better understand the experiences of cases, we examined how perceptions and attitudes differed by demographics, language, and test site, and we assessed differences in sharing of and willingness to share the different types of information that are solicited during contact tracing.

## Methods

### Participants

Eligible participants included those 18 years and older who tested positive for COVID-19 at one of three collaborating testing providers during the periods indicated: Charles R. Drew University of Medicine and Science (CDU)/Martin Luther King Jr., Outpatient Center (MLK-OPC)/Los Angeles County Fire Department, April 2020–January 2021; Kedren Community Health Center (Kedren Health), June 2020–July 2021; and Shared Harvest Foundation (Shared Harvest), April 2020–February 2021 or in August 2021. CDU/MLK-OPC operated COVID-19 testing on the CDU campus and at MLK-OPC. Kedren Health, a federally qualified health center (FQHC), provided testing on site, whereas Shared Harvest conducted mobile testing at various community sites. These three specific collaborating sites were chosen because they accounted for the majority of free public testing COVID-19 testing in South LA for residents during the first year of the pandemic. All research materials were available in English and Spanish. The Institutional Review Board at CDU approved the study and HIPAA waiver.

### Recruitment

Study recruitment occurred between June and November 2021 using various modalities. All COVID-19 testing sites provided the study team with contact and demographic (age, gender, race/ethnicity) information for individuals who had tested positive at their sites. Kedren Health and Shared Harvest representatives initially contacted potential participants through text and email, including an invitational flyer with a general study URL link. The URL directed potential participants to REDCap,^42^ a secure web-based platform to complete the study screener and, if eligible, provide consent and complete the survey. Because MLK-OPC’s site involved a partnership with CDU, the study team made the first study contact with these potential participants.

The study team emailed and sent text messages with individualized URLs for the screener, consent, and study surveys to the potential participants from all three collaborating testing providers. For Kedren Health and Shared Harvest, individual invitations were sent to those that had not responded to the initial invitation, while for MLK-OPC, initial invitations were sent to those testers who had agreed to future contact after being informed of their positive COVID-19 test result. Study staff made phone calls to participants who did not respond to repeated text and email invitations and those whose email information was the same as the others tested. Interested participants contacted by phone were either consented and surveyed by research staff or resent the URL to complete the survey. All study participants received $25 gift cards as compensation for completing the survey.

### Survey Instrument

A review of the literature revealed a lack of validated questions related to experiences with COVID-19 contact tracing. Hence, survey questions were adapted from a Pew Research Center report^43^ and developed from contact-tracing scripts used by the LAC-DPH, and refined with feedback from local community leaders.

The final research survey instrument included questions regarding COVID-19 disease experience (e.g., severity); the contact-tracing process; satisfaction with the public health official/contact tracer; perceptions of information sharing; and level of comfort with sharing information with public health officials/contact tracers.

#### Perceptions of contact-tracing experience and satisfaction with the contact tracer

Participants were asked to rate their overall contact-tracing experience using a four-point Likert scale (poor, fair, good, excellent). To characterize their experience with the tracer, participants were asked, “How would you describe the person’s manner and way of treating you?” Response options were Friendly, Respectful, Indifferent, Caring, Stigmatizing, Rude, Rushed, and Other, with the option to select multiple responses. Participants shared their perspectives on whether they thought the tracer cared about their health. Additional questions included: “How well did the public health official/contact tracer address any questions or concerns that you had?” and “How well did the public health official/contact tracer provide you with information about COVID-19 infection and the course of the illness?”

#### Comfort and trust with sharing information

Level of comfort and trust with sharing information and contacts were measured by asking respondents how much they believed the shared data would “benefit their community and society,” “be used only for public health purposes,” and “remain confidential.” Response options were Not at all, Somewhat, and A lot. Additionally, participants indicated the types of contact-related information they were asked to share, whether they shared the requested information, and their level of comfort with sharing it. Responses were scored on a four-point Likert scale (1: Not at all comfortable–4: Very comfortable).

### Data Quality Assurance

To avoid receiving multiple completed surveys from the same person, a URL that could only be used once was sent to each unique phone number or email provided to the team. However, often testers from the same household provided the same contact information (i.e., phone number, email) during their visit. Additionally, submissions could not be limited when collaborating agencies sent the initial invitations using general URL links. Consequently, in 599 cases, the individual responding to the survey was not the individual on the list provided by the collaborating agency. Since COVID-19 testing was not verified for these individuals, their responses were excluded from analysis. In 299 of these cases, the contact information provided for compensation was repeated on another survey, and variations of the same name were used, leading the study team to suspect they were duplicate submissions. These surveys were largely empty (90–100% unanswered questions), so the impact of their removal on the results was likely minimal. An additional 20 duplicate surveys that were submitted by an individual who had already responded to the survey (based on name and contact information) were also excluded from analysis.

### Analysis

Data analysis was conducted using SAS 9.4. For descriptive statistics, means and proportions (frequencies) were reported. We used chi-square tests to assess perception of contact tracer, contact tracer caring, and assumptions about use of information by testing site, gender, race, age, survey language, response method, and language used with the contact tracer. We used *t*-tests to examine variation in the overall experience with the contact tracer across these same factors. The value of *p* ≤ 0.05 were considered statistically significant.

## Results

The study team attempted to contact 11,718 patients, and obtained completed surveys from 1,413 unique individuals, for a total response rate of 12%. Phone calls resulted in the highest response rates, at close to 20% on first phone contact (Supplementary Table S1). Table 1 describes participants by the three recruitment sites. Overall, participants were majority female (63%), Hispanic/Latino/a/x/e (78%), of ages 18–40 (67%), and completed the survey in English (76%). A large majority reported mild-to-moderate COVID-19 symptoms (72%). Even though 3% reported being hospitalized for COVID-19, 20% of those reporting severe COVID-19 symptoms did not seek medical care. Three-quarters of respondents reported being contacted by the LAC-DPH about their positive COVID-19 result (Table 2), with 57% reporting contact within the first 48 h of receiving the result. A small minority (10%) reported being offered compensation by LAC-DPH for communicating with the tracer.

**Table 1.** Characteristics of Study Population-Testers with a Positive COVID-19 Diagnosis in South Los Angeles

Category	Overall *N* = 1413	MLK/OPC *N* = 571	Kedren *N* = 795	Shared Harvest *N* = 47
	**%**	**%**	**%**	**%**
Gender				
Male	37	39	35	41
Female	63	61	65	59
Race				
Black or African American	15	6	20	57
Hispanic, Latino or Latinx	78	89	73	22
White	3	2	3	15
Asian	2	1	2	4
Other	2	1	2	2
Age				
18–30	43	44	44	19
31–40	24	24	25	26
41–50	17	19	16	21
51–60	10	9	10	21
61+	5	4	5	13
Language				
English	76	70	79	89
Spanish	24	30	21	11
How responded				
Text	32	40	25	38
Email	21	16	25	0
Phone	27	44	14	49
Flyer	20	0	35	13
How severe were your COVID illness symptoms?				
I was in the ICU	1	1	1	0
I was hospitalized but not in the ICU	2	3	1	4
I went to the ER/urgent care but was not hospitalized	5	5	5	4
They were severe but I did not seek medical care	20	19	22	18
They were moderate	36	36	37	25
They were mild	36	37	35	50

**Table 2.** Logistics of Case Investigation/Contact-Tracing Experiences among Testers with a Positive COVID-19 Diagnosis

	Percent	Frequency*
Did anyone from the county/public health department attempt to contact you about your positive COVID-19 test?		
Yes	78	1093
No	16	218
I don’t know	6	85
How long after you received the results of your first positive COVID-19 test did they first try to reach you?		
Within 24 h	21	224
Within 24–48 h	36	394
Within 3–5 days	27	295
Within 6–7 days	6	67
More than 7 days	5	51
I don’t remember	6	61
Were you offered any compensation for communicating with them?^a^		
Yes	10	110
No	90	973
If English is not your primary language, did you talk to an interpreter or did the public health official talk to you in your preferred language?^b^		
Yes	32	334
No	13	136
Not applicable, English is my primary language	54	561
Were you asked to use the CA Notify App^a^		
Yes	16	164
No	84	834

^a^ Of those contacted.

^b^ Of those that spoke with a contact tracer.

*Other differences in totals are due to missing data.

### Contact-Tracing Perceptions

Participants rated contact tracers positively, with 91% affirming at least one positive attribute and 83% agreeing that the tracer demonstrated caring (Table 3). Just 12% endorsed negative attributes. Participants were less likely to strongly endorse positive statements about the contact-tracing process, including statements such as contact tracing “would benefit society” (52%), “be used only for public health purposes” (50%), and “remain confidential” (48%). Nevertheless, they rated their overall experience with contact tracing as positive, with an average mean of 3.1 (“good”).

**Table 3.** Perceptions of Contact Tracer (CT) by Demographics Among Testers with a Positive COVID-19 Diagnosis

	Affirmed positive attributes of CT	Affirmed negative attributes of CT	Sensed CT cared	Assumed info. provided would benefit society	Assumed info. provided would only be used for public health purposes	Assumed info. provided would remain confidential	Overall experience with CT (Scale 1–4)
	%	%	%	%	%	%	mean ± SD
Total	91	12	83	52	50	48	3.1 ± 0.8
Testing Site							
MLK/OPC	92	10	86	57^a^	52	52	3.2 ± 0.8
Kedren	90	14	80	47	47	46	3.1 ± 0.8
Shared Harvest	88	12	76	64	62	47	2.9 ± 0.8
Overall	91	12	83	52	50	48	3.1 ± 0.8
Gender							
Male	90	13	83	54	51^a^	48	3.1 ± 0.8
Female	92	12	83	51	49	49	3.1 ± 0.7
Overall	91	12	83	52	50	49	3.1 ± 0.8
Race							
Black or African American	88	14	83	57	53^a^	53	3.1 ± 0.9
Hispanic, Latino or Latinx	91	12	83	51	49	48	3.1 ± 0.7
White	92	16	88	58	53	45	3.1 ± 0.9
Asian	92	8	85	55	50	41	3.2 ± 0.7
Other	100	6	76	52	39	48	3.2 ± 0.7
Overall	91	12	83	52	50	48	3.1 ± 0.8
Age							
18–30	90	13	82	46^a^	49	44	3.1 ± 0.8
31–40	91	11	81	49	47	49	3.1 ± 0.8
41–50	89	14	82	62	56	59	3.1 ± 0.8
51–60	97	6	92	61	51	46	3.3 ± 0.7
61+	93	11	82	58	44	49	3.1 ± 0.8
Overall	91	12	83	52	50	48	3.1 ± 0.8
Survey language							
English	91	12	82	49^a^	50	47^a^	3.1 ± 0.8
Spanish	91	12	84	62	50	55	3.2 ± 0.7
Overall	91	12	83	52	50	48	3.1 ± 0.8
How responded							
Text message	94^a^	10	85	46^a^	45^a^	47^a^	3.1 ± 0.7
Email	92	12	81	39	46	40	3.1 ± 0.8
Phone call	89	14	84	68	57	58	3.1 ± 0.8
Flyer	88	15	78	48	52	45	3.2 ± 0.8
Overall	91	12	83	52	50	48	3.1 ± 0.8
Whether spoke with contact tracer in preferred language							
Yes	92	11	87	59^a^	52	57^a^	3.2 ± 0.7
No	94	9	81	59	56	50	3.1 ± 0.7
Not applicable, English is my primary language	90	14	81	48	51	46	3.1 ± 0.8
Overall	91	12	83	53	52	50	3.1 ± 0.8

^a^ Indicates statistically significant different between groups.

Tests for differences in perceptions of the process yielded few significant differences and no clear patterns (Table 3). Females’ perceptions that contact-tracing information would benefit society (51%) or be used solely for public health purposes (49%) were somewhat lower than males’ (54% and 51% respectively), but only the latter was statistically significant. Mean scores for the overall process ranged between 2.9 and 3.2 (“good”), regardless of group, with no meaningful differences observed.

Several differences were noted between participants from different testing sites (Table 3). Participants from MLK/OPC were more likely to report that their tracer cared (86%) compared with those from Kedren Health (80%) or Shared Harvest (76%). However, participants from Shared Harvest were most likely to assume the information would benefit society (64%) and be used only for public health purposes (62%), compared with 57% and 52% for MLK/OPC, and 47% and 47% for Kedren Health.

### Differences in Language

A notable group difference observed was in the language used for communicating with the contact tracer. Participants whose primary language was *not* English were *less* likely to indicate the tracer cared (81% vs 87%) or that it would remain confidential (50% vs. 57%) if they were unable to speak with a tracer through an interpreter or in their preferred language, although only the last difference was statistically significant.

### Sharing Information

Participants most frequently reported being asked by the tracer about communal housing situations, followed by locations visited outside California, and their employment (Table 4). For all types of information, at least 76% of participants reported sharing the information requested, and the percentage sharing varied little by type of information (76–85%). Notable differences in comfort level of sharing information were observed between respondents who were requested to share information compared with those who responded hypothetically because they were not asked or did not speak to a contact tracer at all. Between the asked and the hypothetical groups, the difference in comfort was the greatest with sharing shared living situation (87% vs 69%) and employment or school information (82% vs 65%).

**Table 4.** Types of Information and Contacts Requested by and Shared with Contact Tracer

	Asked about N = 1092		Shared (if asked)	Felt somewhat or very comfortable sharing (if asked)	Hypothetically would feel somewhat or very comfortable sharing
	%	#	%	%	%
Shared living situation (if applicable)	77	53	79	87	69
Your employment or school	52	545	83	82	65
Your supervisor’s contact information	21	222	83	61	59
Places you had recently visited outside California and the United States	55	588	81	78	71
Other types of places that you may have visited in California.	47	504	83	78	70
The names of people you may have been in physical contact with	51	531	85	60	62
Location data from your cellphone	15	159	76	38	42

**FIG. 1.**
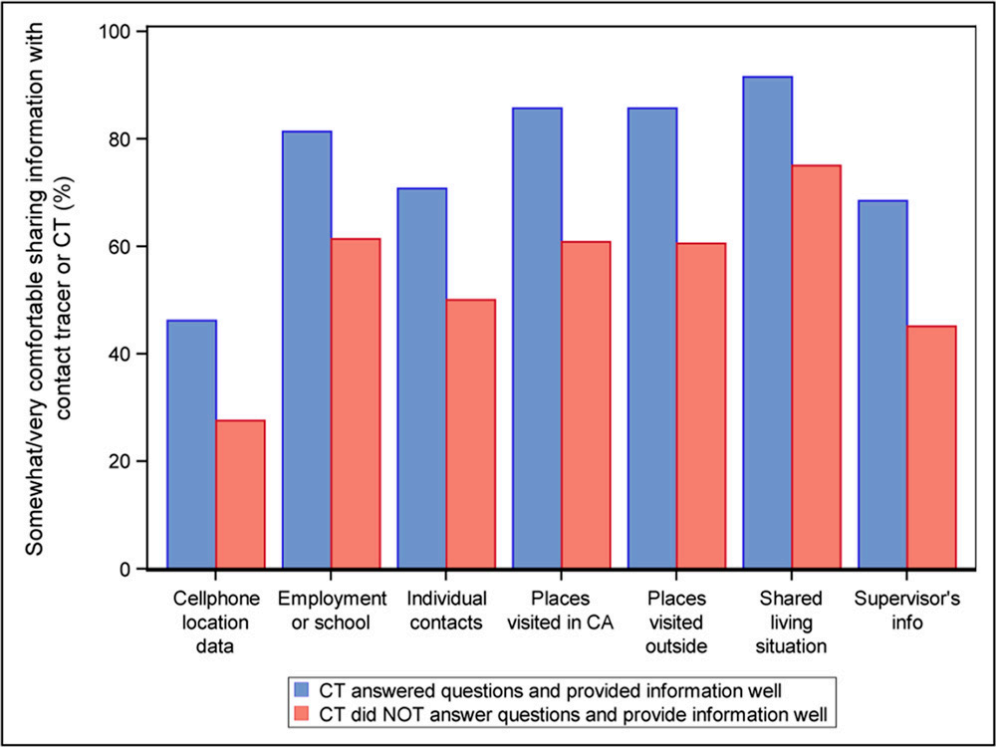
Comfort sharing information with contact tracer by rating of experience with them.

Perception of the tracer and participants’ willingness to share information were associated (Fig. 1). Respondents who believed the tracer did a good job of answering questions and providing information about COVID-19 and those who felt the tracer cared reported feeling more comfortable sharing all information types compared with those who felt the tracer did a poor job in these areas. The percentage of respondents that were comfortable sharing information was 15% to 25% higher among those with favorable views of the contact tracer compared with those with unfavorable views.

## Discussion

Contact-tracing efforts were positively received in this largely Hispanic/Latino and African American/Black sample who tested COVID-19 positive in under-resourced areas of Los Angeles County, which is remarkable given the well-established mistrust of health care and governmental organizations among many in these groups.^30,44,45^ The culturally and linguistically tailored and serviced-oriented approach used to conduct contact tracing and case investigation by LAC-DPH likely contributed to this high level of acceptability, which may have been reinforced by empathetic and trustworthy tracers who could provide helpful disease information. In addition to eliciting potential contacts, tracers were tasked with sharing information on COVID-19 illness management, transmission prevention, and local services and resources, and with answering questions.

Cooperation with this public health initiative likely reflected the rapport, trust, and comfort level between participants and tracers. Positive perceptions of the tracers and their competence indicate the importance of culturally competent and comprehensive training programs. Far fewer respondents endorsed positive statements about the likelihood of their information remaining confidential, being used for public health purposes, and benefiting society than about the tracers. Little variation was observed across demographics in participants’ perceptions of the tracer and their interaction, except those surveyed in Spanish consistently reported more positive perceptions. However, participants responded more unfavorably to hypothetical questions about queries for specific information than when queried about specific information that tracers had actually requested of them. Those responding to hypothetical questions were more likely to have had little or no interaction with a tracer. Hence, their negative responses may have reflected preexisting mistrust or the tracer not having the opportunity to educate and build rapport with them.

Participants who communicated with the tracer in their preferred language or through an interpreter were more likely to endorse positive perceptions about the tracer and how their information would be used compared with those who did not. This may reflect the importance of linguistic concordance to creating comfort, quality communication, and confidence in these interactions.^46^ Being offered compensation for communication with a tracer may also increase positive perceptions; however, we did not examine whether the perceptions differed for the 10% who were compensated. As compensation was only offered for a short period, it would not be possible to assess whether any differences in perception observed reflected the compensation or the specific time of the pandemic in which it was offered.

Over 20% endorsed severe COVID-19 symptoms and never sought medical care. It is documented that under-resourced minorities faced disproportionate burden, as evidenced by higher COVID-19 mortality rates^47^ and treatment inequities for severe COVID-19 illness.^48^ Additionally, high rates of COVID-19,^27^ poverty,^49,50^ immigration policies and enforcement,^51^ lack of education and poor health literacy,^52,53^ and concerns about intrusion by child welfare agencies^54,55^ increase the likelihood populations similar to those studied here will have concerns about engaging with health care agencies. Contact tracers and providers who engage cases and contacts authentically, acknowledge and address sources of mistrust, and offer both clear guidance and needed resources may help overcome these concerns.^15,41^

Many consider the collaborating agencies to be trusted, community-based resources, and two are safety net providers. Despite differences by testing site, participants expressed at high levels that their tracer cared. Moreover, Shared Harvest, which conducted “pop-up” testing may have been perceived as solely for testing and public health efforts, compared with MLK/OPC and Kedren Health, whose other services are more evident. Participants may have also received additional resources from the collaborating agencies, including referrals, care for COVID-19 or other conditions, and information on best practices for COVID-19 recovery.^56^ Success of contact-tracing methods for targeted communities may be contingent on strong partnerships between public health departments and safety net health systems. Therefore, it is important that public health agencies and leaders implement innovative messaging campaigns and community engagement strategies. This may include collaboration with community leaders, administrators at collaborating testing sites, and health care providers to increase the trustworthiness, cultural and linguistic sensitivity, and humility of public health activities.

### Implications

Contact-tracing programs and trainings should focus on rapport building between participant and tracer, identifying sources of mistrust, and determining outreach preferences for different groups.^57^ The Los Angeles County Departments of Public Health and Health Services provided motivational interviewing and cultural competency/cultural humility training to contact tracers. This is recommended to increase the willingness to provide sensitive information, address concerns, and encourage adherence to COVID-19 contact tracing and isolation/quarantine protocols among cases and their potential contacts.^12,58–60^ While less true later in the pandemic, at the time of data collection, a COVID-19 diagnosis may have been stigmatizing and had negative implications for job security and ability to care for loved ones, especially among under-resourced groups who may have lacked paid leave. Tracers should offer information and referrals to ease these concerns, and to address the physical and mental health challenges, housing and social support needs, and health care access barriers raised by cases and contacts.^58^ Finally, concerns about data security and uses of the solicited information were high and willingness to share cellphone location data were low; hence, perceptions and acceptability of digital technology for contact tracing warrant further study in racial/ethnic and other minoritized groups.^61^ Even in the absence of digital tracing, technology can be used to make contact tracing more efficient, such as through automated routines for scheduling, messaging, calling, and tracking attempts to reach cases and contacts.

Community health worker (CHW) programs specializing in neighborhood contact tracing in under-resourced communities may expand the acceptability and reach of contact tracing in these populations for COVID-19 and future epidemics.^62^ CHWs can be well equipped to mitigate COVID-19 risks and generally have expertise in the lived experience, social environment, and cultural norms of cases and contacts. Development of such programs may require collaborations between health care systems, local community organizations (e.g., advocacy groups), and other social institutions (e.g., churches).

Future research may explore how public health officials can assess the role of health care status and interactions with public health and health care delivery systems in the perceptions of case investigation and contact tracing for other racial and ethnic populations, such as Asians, Pacific Islanders, and Native Hawaiians. Comparative data from other jurisdictions are needed to determine whether these findings are specific to South Los Angeles or to public health systems that have prioritized community centered, structurally competent, and culturally informed training of contact tracers.

### Limitations

One study limitation is reduction in sample size from excluding participants who responded to an individualized URL intended for another individual and those who submitted fraudulent submissions through the general URL link. Our sample is also limited to those who independently sought out a COVID-19 test, received study messaging, and responded. This may have led to biased data as participants may have been more engaged in their health and well-being, expressed more positive perceptions, and have greater openness about information sharing than nonparticipants. In addition, multiple individuals in the same household could have been surveyed if each had a positive test result, and it is possible that individuals encouraged one another to participate, increasing selection bias. Although use of incentives and repeated contact attempts may have limited this selection bias, bias due to nonresponse remains a concern. Low response rates are consistent with remote survey research of this type, such as the 2018 phone-based LA County Health Survey that reports an 11% participation rate,^63^ and many individuals reported fatigue with discussions of COVID-19.

Many participants received calls from both LAC-DPH tracers and providers from Kedren Health and Shared Harvest agencies regarding their positive result and may have been unable to distinguish the two calls when responding to the survey. In the survey, we specifically asked about contact with a “public health official” to reduce confusion. However, tracers and providers from Kedren or Shared Harvest would not have asked for specific types of information, so participants who mixed up multiple calls may have been more likely to indicate that they had not been asked for information and would have responded to the hypothetical questions.

Additionally, recall bias may be an inherent study limitation due to the length of time between COVID-19 testing and responding to a study interviewer, despite participants having verified COVID-19 testing records. Additionally, we note that participants were assessed regarding their experiences with contact tracing and not their adherence to isolation or quarantine guidelines following their COVID-19 test/results. Finally, participants surveyed in English identified as the full range of race/ethnicities, while those surveyed in Spanish nearly always identified as Hispanic/Latino, potentially confounding the associations with language. Despite these limitations, study findings strongly contribute to the current knowledge base that has a paucity of information on COVID-19 contact-tracing perceptions.

## Conclusion

Study findings showed that contact-tracing efforts were positively received among this predominantly Hispanic/Latino and African American/Black sample. This may reflect the well-trained and culturally competent tracers working for LAC-DPH, who delivered adequate information on both COVID-19 infection and needed resources. Policy protections for workers and enhanced privacy protections may reduce reluctance to report information on workplaces and personal contacts for study participants and marginalized communities. Investments in linguistically concordant services may increase effectiveness of this public health intervention. Future research should examine factors affecting levels of comfort with sharing data with public health officials. To foster positive and effective interactions between participants and tracers, public health entities should emphasize community trust building and engagement in their design and delivery of contact-tracing programs.

## Abbreviations Used

CDU: Charles R. Drew University of Medicine and Science
CHW: Community health worker
COVID-19: Coronavirus disease 2019
FQHC: Federally qualified health center
HIPAA: Health Insurance Portability and Accountability Act
Kedren Health: Kedren Community Health Center
LAC-DPH: Los Angeles County Department of Public Health
MLK-OPC: Martin Luther King Jr., Outpatient Center
Shared Harvest: Shared Harvest Foundation
