# Supplementary material for: An Examination of Responses to COVID-19 Contact-Tracing Efforts in Black/African American and Hispanic/Latinx Communities of Los Angeles
Source: Health Equity. 2024 Aug 7;8(1):493–504. doi: 10.1089/heq.2023.0243 (PMC11347877; doi:10.1089/heq.2023.0243)
Supplement: Supplementary Table S1 [file heq.2023.0243_supplement_table.pdf]

Table S1. Response rates to outreach efforts by attempt type and number

	Flier (n=8523)				
Responded to flier	3.6%				
Began survey (of responded)	100.0%				
Completed survey (of began)	87.5%				
	Text1 (n=10992)	Text2 (n=10403)	Text3 (n=9892)	Text4 (n=9696)	
Switched to Spanish contact	66				
Disconnected	2.7%				
Opt out	2.5%				
Responded	4.9%	2.7%	1.7%	2.8%	
Interested in survey (of responded)	91.8%	85.8%	87.3%	51.2%	
Completed consent (of interested)	60.9%	58.0%	49.5%	47.6%	
Began survey (of consented)	95.5%	93.8%	94.2%	84.9%	
Completed survey (of began)	95.3%	96.2%	98.0%	94.1%	
	Email1 (n=7884)	Email2 (n=7625)	Email3 (n=7158)	Email4 (n=7014)	
Switched to Spanish contact	53				
Responded	2.2%	1.1%	1.0%	1.1%	
Interested in survey (of responded)	95.3%	91.7%	93.0%	89.4%	
Completed consent (of interested)	66.5%	67.5%	72.7%	59.2%	
Began survey (of consented)	90.8%	98.1%	91.7%	93.5%	
Completed survey (of began)	97.0%	98.1%	100.0%	95.4%	
	Call1 (n=2738)	Call2 (n=1083)	Call3 (n=317)	Call4 (n=37)	Call5 (n=6)
Requested Spanish speaker	13.3%	4.4%	5.0%	3.2%	0.0%
Wrong number	4.2%	3.0%	1.1%	0.0%	0.0%
Voicemail full or no Voicemail	3.6%	12.3%	8.9%	9.7%	0.0%
No connection	9.6%	2.2%	5.0%	3.2%	0.0%
Voicemail	27.7%	55.4%	66.8%	58.1%	33.3%
Declined	20.9%	18.8%	8.6%	6.5%	0.0%
Requested callback	5.1%	21.9%	26.8%	38.7%	66.7%
Completed survey	18.6%	18.9%	19.6%	32.3%	100.0%
Sent survey link	2.7%	7.2%	0.0%	9.4%	50.0%
