# Supplementary material for: An Examination of Responses to COVID-19 Contact-Tracing Efforts in Black/African American and Hispanic/Latinx Communities of Los Angeles
Source: Health Equity. 2024 Aug 7;8(1):493–504. doi: 10.1089/heq.2023.0243 (PMC11347877; doi:10.1089/heq.2023.0243)
Supplement: Supplementary Data S1 [file heq.2023.0243_experience_with_contacttracing_survey.pdf]

Thank you for agreeing to participate in our study. We appreciate your contribution.

Remember, your responses will be kept confidential, so please be honest. If you are not sure of an answer, just give your best guess.

When you received your positive test result, did you believe you had COVID-19?	◯ Yes<br>◯ No
What made you believe you had COVID-19? (Select all that apply)	☐ COVID-19 Test Result<br>☐ COVID-19 Symptoms<br>☐ Family/Friends
Why didn't you believe you had COVID-19? (Select all that apply)	☐ It doesn't exist<br>☐ COVID-19 test was a false positive<br>☐ I didn't have any symptoms
Did you experience COVID-19 symptoms?	◯ Yes<br>◯ No
How severe were your COVID illness symptoms?	◯ I was in the ICU<br>◯ I was hospitalized but not in the ICU<br>◯ I went to the ER/urgent care but was not hospitalized<br>◯ They were severe but I did not seek medical care<br>◯ They were moderate<br>◯ They were mild
Around the time you first tested positive for COVID, were you living in a congregate living facility? A congregate living facility is a living facility that offers living, sleeping, food and other needs for residents (including jail, prison, or other detention centers).	◯ Yes<br>◯ No
Were you able to identify when and where you became infected with COVID-19?	◯ Yes<br>◯ No

Contract tracing

Did anyone from the county/public health department attempt to contact you about your positive COVID-19 test?

- ◯ Yes
- ◯ No
- ◯ I don't know

How did they attempt to reach you? (Select all that apply)

- ☐ Phone (direct)
- ☐ Left voice mail
- ☐ Email
- ☐ Text message
- ☐ Came to my home
- ☐ Other

Please describe any other way that someone from the county/public health department tried to contact you?

____

How long after you received the results of your first positive COVID-19 test did they first try to reach you?

- ◯ Within 24 hours
- ◯ Within 24-48 hours
- ◯ Within 3-5 days
- ◯ Within 6-7 days
- ◯ More than 7 days
- ◯ I don't remember

How long after you received the results of your first positive COVID test did you first communicate with someone from the health department?

- ◯ Within 24 hours
- ◯ Within 24-48 hours
- ◯ Within 3-5 days
- ◯ Within 6-7 days
- ◯ More than 7 days
- ◯ I don't remember
- ◯ I never talked to anyone from the county/public health department

How did you communicate with them? (Select all that apply)

- ☐ In person
- ☐ Via phone
- ☐ Via text
- ☐ Via email

Were you offered any compensation for communicating with them?

- ◯ Yes
- ◯ No

What services or information did they offer to you? (Select all that apply)

- ☐ Information about COVID disease and treatment
- ☐ Information about quarantine restrictions
- ☐ Requests for participation in contact tracing
- ☐ Information about temporary housing for people in need
- ☐ Information about other community resources (e.g. food, mental health)

How likely, if at all, would you be to speak with a public health official /contact tracer if they contacted you by phone or text message to speak with you about the COVID-19 outbreak?

- ◯ Very likely
- ◯ Somewhat likely
- ◯ Not too likely
- ◯ Not at all likely

How likely, if at all, would you be to speak with a public health official /contact tracer if they showed up at your residence to speak with you about the COVID-19 outbreak?

- ◯ Very likely
- ◯ Somewhat likely
- ◯ Not too likely
- ◯ Not at all likely

Were you asked about each of the following?

	Yes	No
Details about your shared living situation	◯	◯
Your employment or school	◯	◯
Your supervisor's contact information	◯	◯
Places you have recently visited outside California and the US	◯	◯
Other types of places that you may have visited in California.	◯	◯
The names of people you may have been in physical contact with	◯	◯
Location data from your cellphone	◯	◯

How comfortable, if at all, did you feel sharing the following with a public health official/contact tracing during the coronavirus outbreak?

Details about your shared living situation	◯ Very comfortable<br>◯ Somewhat comfortable<br>◯ Not too comfortable<br>◯ Not at all comfortable
Your employment or school	◯ Very comfortable<br>◯ Somewhat comfortable<br>◯ Not too comfortable<br>◯ Not at all comfortable
Your supervisor's contact information	◯ Very comfortable<br>◯ Somewhat comfortable<br>◯ Not too comfortable<br>◯ Not at all comfortable
Places you have recently visited outside California and the US	◯ Very comfortable<br>◯ Somewhat comfortable<br>◯ Not too comfortable<br>◯ Not at all comfortable
Other types of places that you may have visited in California.	◯ Very comfortable<br>◯ Somewhat comfortable<br>◯ Not too comfortable<br>◯ Not at all comfortable
The names of people you may have been in physical contact with	◯ Very comfortable<br>◯ Somewhat comfortable<br>◯ Not too comfortable<br>◯ Not at all comfortable
Location data from your cellphone	◯ Very comfortable<br>◯ Somewhat comfortable<br>◯ Not too comfortable<br>◯ Not at all comfortable

How comfortable, if at all, would you be sharing the following with a public health official/contact tracer during the COVID-19 outbreak?

Details about your shared living situation	◯ Very comfortable<br>◯ Somewhat comfortable<br>◯ Not too comfortable<br>◯ Not at all comfortable
Your employment or school	◯ Very comfortable<br>◯ Somewhat comfortable<br>◯ Not too comfortable<br>◯ Not at all comfortable
Your supervisor's contact information	◯ Very comfortable<br>◯ Somewhat comfortable<br>◯ Not too comfortable<br>◯ Not at all comfortable
Places you have recently visited outside California and the US	◯ Very comfortable<br>◯ Somewhat comfortable<br>◯ Not too comfortable<br>◯ Not at all comfortable
Other types of places that you may have visited in California.	◯ Very comfortable<br>◯ Somewhat comfortable<br>◯ Not too comfortable<br>◯ Not at all comfortable
The names of people you may have been in physical contact with	◯ Very comfortable<br>◯ Somewhat comfortable<br>◯ Not too comfortable<br>◯ Not at all comfortable
Location data from your cellphone	◯ Very comfortable<br>◯ Somewhat comfortable<br>◯ Not too comfortable<br>◯ Not at all comfortable

Did you share the following information?

	Yes	No
Details about your shared living situation	○	○
Your employment or school	○	○
Your supervisor's contact information	○	○
Places you have recently visited outside California and the US	○	○
Other types of places that you may have visited in California.	○	○
The names of people you may have been in physical contact with	○	○
Location data from your cellphone	○	○

How acceptable is it for the personal information that may be collected during contact tracing during the COVID-19 outbreak to be available for use by

Researchers who study infectious diseases

◯ Very acceptable
◯ Somewhat acceptable
◯ Somewhat unacceptable
◯ Very unacceptable

Health insurance companies

◯ Very acceptable
◯ Somewhat acceptable
◯ Somewhat unacceptable
◯ Very unacceptable

Pharmaceutical companies

◯ Very acceptable
◯ Somewhat acceptable
◯ Somewhat unacceptable
◯ Very unacceptable

State and local elected officials

◯ Very acceptable
◯ Somewhat acceptable
◯ Somewhat unacceptable
◯ Very unacceptable

Companies conducting clinical trials

◯ Very acceptable
◯ Somewhat acceptable
◯ Somewhat unacceptable
◯ Very unacceptable

How well did the public health official/contact tracer address any questions or concerns that you had?

◯ Very well
◯ Somewhat well
◯ Not too well
◯ Not well at all

How well did the public health official/contact tracer provide you information about COVID-19 infection and the course of the illness?

◯ Very well
◯ Somewhat well
◯ Not too well
◯ Not well at all

Did you sense that the person cared about you and your health?

◯ Yes
◯ No

Why did you feel the person did not care about you or your health?

____

How would you describe the person's manner and way of treating you? (Select all that apply)

☐ Friendly
☐ Respectful
☐ Indifferent
☐ Caring
☐ Stigmatizing
☐ Rude
☐ Rushed
☐ Other

Please describe any other way that the person treated you.

____

If English is not your primary language, did you talk to an interpreter or did the public health official talk to you in your preferred language?

◯ Yes
◯ No
◯ Not applicable, English is my primary language

Individual contacts

You said you shared contacts with the public health official/contact tracer. Were there people whose names you didn't share?

◯ Yes
◯ No
◯ I don't remember

Indicate the reason or reasons you did not share the names of your individual contacts. (Select all that apply)

☐ I had already informed the person
☐ The person was undocumented
☐ The person had legal or child custody issues
☐ The person would see it as a privacy violation
☐ The person had COVID before me
☐ I did not have any contact information for the person
☐ The person was a coworker and I did not want to lose my job
☐ The person was a patient and I did not want to lose my job
☐ I didn't want them to know I had a positive COVID test
☐ Other

Please specify the other reason(s) you did not share your individual contact(s).

____

Would you have been willing to provide contact information from the contact tracer to your individual contacts and encourage them to get in touch?

◯ Yes
◯ No

Did you tell your individual contacts about your COVID test result yourself?

◯ Yes
◯ No

How did you inform them? (Select all that apply)

☐ Phone
☐ In-Person
☐ Text
☐ Email
☐ Other

How else did you tell them?

____

How did your contacts react when they were contacted by the health department after you communicated with a contact tracer? (Select all that apply)

☐ Anger
☐ Fear
☐ Gratitude
☐ Indifference (They didn't care)
☐ Embarrassment
☐ Other
☐ I don't know
☐ Not applicable

How did your contacts react when you told them directly that you had COVID-19? (Select all that apply)

☐ Anger
☐ Fear
☐ Gratitude
☐ Indifference (They didn't care)
☐ Embarrassment
☐ Other
☐ I don't know
☐ Not applicable

How much did you trust that the information you provided to the Contact Tracer would be used in the following ways?

Beneficial for my community and society

◯ A lot
◯ Somewhat
◯ Not at all

Only for public health purposes

◯ A lot
◯ Somewhat
◯ Not at all

Would remain confidential

◯ A lot
◯ Somewhat
◯ Not at all

How much do you trust that the information people provide to Contact Tracers would be used in the following ways?

Beneficial for my community and society

◯ A lot
◯ Somewhat
◯ Not at all

Only for public health purposes

◯ A lot
◯ Somewhat
◯ Not at all

Would remain confidential

◯ A lot
◯ Somewhat
◯ Not at all

Consequences of contact tracing

Did you experience any negative consequences related to contact tracing?

◯ Yes
◯ No

What areas of your life did these negative experiences affect? (Select all that apply)

☐ Your employment
☐ Your housing
☐ Your legal status
☐ Your custody of children
☐ Your ability to travel
☐ Other

What other areas of your life were negatively affected?

____

Rate your overall experience with contact tracing

◯ Excellent
◯ Good
◯ Fair
◯ Poor

How might the contact tracing experience be improved?

____

Were you asked to use the CA Notify App?

◯ Yes
◯ No

Did you download it to your phone and use it?

◯ Yes
◯ No

Did you use the LA Public Health COVID contact tracing hotline?

◯ Yes
◯ No

Other contact tracing efforts

Were you contacted by anyone else for contact tracing?

◯ Yes
◯ No

Who contacted you?

☐ Mobile app-based contact
☐ Employer
☐ University or School
☐ Congregate living
☐ Police
☐ Faith community
☐ Coach
☐ Other

Who else contacted you for contact tracing?

____

Did you respond to their requests for contact tracing?

◯ Yes
◯ No

How long after you received the results of your first positive COVID-19 test did they first try to reach you?

◯ Within 24 hours
◯ Within 24-48 hours
◯ Within 3-5 days
◯ Within 6-7 days
◯ More than 7 days
◯ I don't remember

How did you communicate with them? (Select all that apply)

☐ In person
☐ Via phone
☐ Via text
☐ Via email

Where you asked about each of the following?

	Yes	No
Details about your shared living situation	○	○
Your employment or school	○	○
Your supervisor's contact information	○	○
Places you have recently visited outside California and the US	○	○
Other types of places that you may have visited in California.	○	○
The names of people you may have been in physical contact with	○	○
Location data from your cellphone	○	○

How comfortable, if at all, did you feel sharing the following with a public health official/contact tracing during the coronavirus outbreak?

Details about your shared living situation	◯ Very comfortable<br>◯ Somewhat comfortable<br>◯ Not too comfortable<br>◯ Not at all comfortable
Your employment or school	◯ Very comfortable<br>◯ Somewhat comfortable<br>◯ Not too comfortable<br>◯ Not at all comfortable
Your supervisors contact information	◯ Very comfortable<br>◯ Somewhat comfortable<br>◯ Not too comfortable<br>◯ Not at all comfortable
Places you have recently visited outside California and the US	◯ Very comfortable<br>◯ Somewhat comfortable<br>◯ Not too comfortable<br>◯ Not at all comfortable
Other types of places that you may have visited in California.	◯ Very comfortable<br>◯ Somewhat comfortable<br>◯ Not too comfortable<br>◯ Not at all comfortable
The names of people you may have been in physical contact with	◯ Very comfortable<br>◯ Somewhat comfortable<br>◯ Not too comfortable<br>◯ Not at all comfortable
Location data from your cellphone	◯ Very comfortable<br>◯ Somewhat comfortable<br>◯ Not too comfortable<br>◯ Not at all comfortable

Did you share the following information?

	Yes	No
Details about your shared living situation	◯	◯
Your employment or school	◯	◯
Your supervisor's contact information	◯	◯
Places you have recently visited outside California and the US	◯	◯
Other types of places that you may have visited in California.	◯	◯
The names of people you may have been in physical contact with	◯	◯
Location data from your cellphone	◯	◯

Did you sense that the person cared about you and your health?

◯ Yes
◯ No

Why did you feel the person did not care about you or your health?

____

How would you describe the person's manner and way of treating you? (Select all that apply)

◯ Friendly
◯ Respectful
◯ Indifferent
◯ Caring
◯ Stigmatizing
◯ Rude
◯ Rushed
◯ Other

Please describe any other way that the person treated you.

____

Would you be interested in participating in additional research efforts from our study group?	◯ Yes<br>◯ No

Please click the submit button to complete the survey.
